# Research Progress of Pyroptosis in Diabetic Kidney Disease

**DOI:** 10.3390/ijms25137130

**Published:** 2024-06-28

**Authors:** Qingqing Fan, Rongxuan Li, Huiting Wei, Weiyue Xue, Xiang Li, Ziyao Xia, Le Zhao, Ye Qiu, Di Cui

**Affiliations:** 1 Department of Physical Education, Hunan University, Changsha 410000, China; fanqingqing@hnu.edu.cn (Q.F.); lirongxuan@hnu.edu.cn (R.L.); w1486776921@hnu.edu.cn (H.W.); s212301567@hnu.edu.cn (W.X.); xiaziyao@hnu.edu.cn (Z.X.); zhaole@hnu.edu.cn (L.Z.); 2 Department of Physical Education, Jiangnan University, Wuxi 214122, China; 8202309001@jiangnan.edu.cn; 3 The State Key Laboratory of Medical Virology, College of Biology, Hunan University, Changsha 410000, China; qiuye@hnu.edu.cn

**Keywords:** pyroptosis, diabetic nephropathy, GSDMs, caspases, NLRP3

## Abstract

Pyroptosis, known as one typical mode of programmed cell death, is generally characterized by the cleaved gasdermin family (GSDMs) forming pores in the cell membrane and inducing cell rupture, and the activation of aspartate-specific proteases (caspases) has also been found during this process. Diabetic Kidney Disease (DKD) is caused by the complication of diabetes in the kidney, and the most important kidney’s function, Glomerular Filtration Rate (GFR), happens to drop to less than 90% of its usual and even lead to kidney failure in severe cases. The persistent inflammatory state induced by high blood glucose implies the key pathology of DKD, and growing evidence shows that pyroptosis serves as a significant contributor to this chronic immune-mediated inflammatory disorder. Currently, the expanded discovery of GSDMs, pyroptosis, and its association with innate immunity has been more attractive, and overwhelming research is needed to sort out the implication of pyroptosis in DKD pathology. In this review, we comb both classical studies and newly founds on pyroptosis, prick off the novel awakening of pyroptosis in DKD, and center on the significance of pyroptosis in DKD treatment, aiming to provide new research targets and treatment strategies on DKD.

## 1. Introduction

The deterioration of kidney function in diabetes is termed diabetic kidney disease (DKD), and the featured index of DKD is the dramatic drop of the glomerular filtration rate (GFR) of glomeruli to less than 90% and even leading to kidney failure, or end-stage renal disease (ESRD), in severe cases. Over the past decade, the incidence and prevalence of DKD have increased dramatically, with a prevalence of 20–40% of the 463 million diabetics worldwide [1,2]. In developed countries, diabetes accounts for 30–50% of ESRD, and DKD has become the leading cause of ESRD worldwide, making it a serious public health problem for the global population, and symptoms are sequentially characterized by proteinuria, glomerular hypertrophy, renal fibrosis, and progressive decline in renal function. Early treatment is effective, whereas kidney failure can only be treated with dialysis and finite kidney transplantation, causing not only a heavy financial burden on healthcare but also an inevitable risk of immune rejection. Hence, the complex pathological mechanisms have remained to be revealed, and novel therapeutic strategies are urgently needed as well [3,4].

Recent evidence indicates that pyroptosis is involved in DKD guiding new directions and therapeutic implications. Pyroptosis, termed for a typical inflammatory pattern of programmed cell death, is generally featured by the diversified gasdermin family (GSDMs, mainly GSDMD/GSDME) generating pores on the plasma membrane to facilitate cell expansion and broad release of pro-inflammatory factors, including interleukin-1β (IL-1β) and IL-18, and the cleavage of GSDMD and pro-inflammatory precursors was executed by cysteinyl aspartate-specific proteinase-1 (caspase-1) in canonical pathways and possible caspase-4/5/11 in non-canonical pathways [5]. Studies have confirmed that high glucose exposure gave rise to NLR Family Pyrin Domain Containing 3 (NLRP3) inflammasome activation, and its mediated pyroptosis in the kidney and the cascading inflammatory results to some extent deciphered the pathogenesis of DKD [6]. Along with the deepening of research, the pyroptosis research scope was sprawling, especially with the progress of findings on GSDMs and pyroptosis mechanisms. Therefore, not only the frontiers in pyroptosis need to be recognized, but also the pathomechanism of pyroptosis in DKD and its therapeutic implications are urgently needed for further study.

In this review, we comb both classical studies and newly founds on pyroptosis, prick off the novel awakening of pyroptosis in DKD, and center on the significance of pyroptosis in DKD treatment, aiming to provide new research targets and treatment strategies on DKD. 

## 2. GSDMs, Inflammasomes, and Caspases in Pyroptosis

The incipient discovery of pyroptosis can be traced back to Friedlander’s study in 1986, in which mouse peritoneal macrophages were killed by the rapid release of cell contents only with short-term exposure to anthrax lethal toxin [7]. Zychlinsky and his team successively observed the caspase-1-dependent inflammatory cell death in the Gram-negative bacterial pathogen Shigella flexneri-infected macrophages in eight years since 1992, and they also proved that caspase-1 activation of IL-1beta and IL-18 were essential in that process [8,9,10]. Later in 2001, Cookson and Brennan proposed the term pyroptosis to describe pro-inflammatory programmed cell death, which was derived from the Greek *pyro*, representing fire or fever, and *ptosis*, standing for a falling [11,12]. It was during that period that this inflammatory programmed cell death had been detached distinctly from traditional non-inflammatory programed cell death, apoptosis. In both canonical caspase-1- and non-canonical casepase-4/5/11-dependent pyroptosis, GSDMD was reported to be cleaved and facilitated its N-terminal domain oligomerization forming pores in cell membrane and inducing cell rupture [13,14]. Additionally, more GSDMs (mainly GSDMB, GSDMC, and GSDME) have been found and proven effective in pyroptosis, and along with GSDMD, GSDMs, and GSDM-mediated pyroptosis involved in the regulation of immunity and multiple diseases, which extended our knowledge scope on pyroptosis and its possible implications on inflammatory disorders [15,16,17,18].

## 3. GSDMs Execute Pore-Forming Function in Pyroptosis

Generally, most GSDMs are structurally linked with the C-terminal repressor domain (RD/CT) and N-terminal pore-forming domain (PFD/NT), functioned in liposome-leakage and pore-forming activity, and when inflammasomes form or other inflammatory stimuli occur, the PFD will be cleaved by each corresponding caspases or proteolytic enzymes and oligomerize to construct large pores in the cell membrane, facilitate inflammatory molecules, and ultimately result in cell swelling and rupture [19]. Up until now, there were 6 (GSDMA-D, GSDME/DFNA5, and DFNB59/PJVK) in humans, and 10 (Gsdma1–3, Gsdmc1–4, Gsdmd, Dfna5, and Dfnb59) in mice macrophages gasdermin homologues possessing resembling N-terminal domain found, and the most focused targets were GSDMD and GSDME among the published studies [20]. Human GSDMD is gnomically located on chr8q24.3, encoding a 52 kDa protein composed of 484 AA and expressed widely in major tissues staying intramolecular autoinhibition in the absence of cleavage by inflammatory caspases. The linker between RD and PFD in GSDMD can be specifically cleaved by activated caspase-1 when ligands of various canonical inflammasomes arise and caspase-4/5/11 when encountered with bacterial lipopolysaccharide (LPS). Direct testimony showed that GSDMD-deficient cells resisted to induce pyroptosis, and synergistically IL-1β release was also diminished [20]. Hence, inhibiting GSDMD directly represses pyroptosis. Additionally, GSDMD-mediated pyroptosis can also be blocked by small molecules targeting the cleavage of GSDMD and its PFD oligomerization, such as necrosulfonamide (NSA), LDC7559, magnesium (Mg^2+^), disulfiram, and succination of GSDMD [21]. The transcriptional regulation of nuclear factor κB (NF-κB) on GSDMD was evidenced in adipocytes, and the hypermethylation of the GSDMD promoter region was also proven to repress its expression in NK92 cells [22,23]. Interferon regulatory factor 2 (IRF2) was confirmed to enhance GSDMD transcription by loss-of-function study in endothelial cells (ECs) and mouse macrophage [24]. Both GSDMD-mediated canonical and non-canonical pyroptosis can be activated directly by LPS, while other chemicals or factors mediate pyroptosis by lytic caspase-3/6/7 [19,25]. Human GSDME, also known as ICERE-1/DFNA5, is gnomically located on chr7P15.3, encoding a 55 kDa protein composed of 496 AA, and its PFD cleavage can be produced by caspase-3 or granzyme B (GZMB) [26]. GSDME was initially identified as a gene implying hearing loss, and later studies reported it in multiple types of tumors [25]. It was not proven until 2020 that caspase-3 cleaving GSDME mediated non-apoptic, non-canonical pyroptosis, and GSDME served as a tumor suppressor, activating pyroptosis and enhancing anti-tumor immunity [27]. In ovarian cancer cells, when GSDME is highly expressed, caspase-3 cleaves it, releasing the N-terminal structural domain to punch holes in the cell membrane, leading to cell swelling, rupture, and death [28]. Unlike other gasdermin family proteins, the N-terminal of GSDMB itself does not induce pyroptosis but promotes caspases-4 activity launching GSDMD cleavage and the non-classical pyroptosis pathway [29]. In HeLa cells, the metabolite α-ketoglutarate (α-KG) can induce pyroptosis via caspase-8 cleavage of GSDMC at Asp240, producing the N-terminal end of GSDMC [30]. GSDMC was transcriptional-enhanced by nuclear-located programmed death ligand 1 (PD-L1) switching tumor necrosis factor-alpha (TNF-α)-induced apoptosis to pyroptosis in cancer cells, and its N-terminal domain was specifically cleaved by caspase-8, with TNFα treatment forming pores on the cell membrane and inducing cell rupture [31]. 

## 4. Inflammasomes and Other Pathogenic Sensor Signals Initiate Pyroptosis

Pyroptosis is initiated by inflammasomes when the cell undergoes cytosolic contamination or perturbation [32]. Inflammasomes, an inflammatory vesicle multiprotein complex assembled with pattern recognition receptors (PPRs), were incipiently reported in innate immunity and widely distributed in immune cells. Later, with the discovery of pyroptosis, especially in the canonical pathway, inflammasomes were elucidated, playing a vital role in initiating caspase-1/GSDMD. PPR, also known as inflammatory vesicle sensor, recognizes extracellular exogenous pathogen-associated molecular patterns (PAMPs) or endogenous damage-associated molecular patterns (DAMPs) mainly by membranal Toll-like receptor (TLR) and C-type lectin receptor (CLR), and intracellularly by RIG-like receptor (RLR), absent in melanoma 2 (AIM2), and NOD-like receptor (NLR) [32]. Once the recognitions of PPRs occur, inflammasomes will be fabricated by PPR auto-oligomerization, pro-caspase-1 (containing a caspase activation and recruitment domain (CARD), and apoptosis-associated speck-like protein containing a CARD (ASC, also containing a pyrin domain (PYD)) [33]. Currently, well-studied inflammasomes are concentrated in the NLR family and PYHIN family. The NLR family, represented by NLRP1, NLRP3, and NLRC4, is structurally composed of carboxy-terminal leucine-rich repeat domain (LRR), a nucleotide-binding domain (NBD), and either a PYD or CARD or both, while the PYHIN family, represented by AIM2, is characterized as possessing a HIN200 in addition to PYD [34,35]. NLRP1 inflammasome, distributed relative limit, was the first to be reported targeting caspase-1 with structurally possessing a unique function-to-find domain (FIIND), and it had been confirmed that the proteasome-mediated degradation of NLRP1 was both necessary and sufficient for NLRP1 activation [36]. The NLRP3 inflammasome consists of three protein subunits, ASC, NLRP3, and pro-caspase-1, and its temporal and spatial formation requires going through at least two procedures, initiation and activation [37]. In mouse macrophages, firstly, for example, when TLRs (e.g., TLR4) recognize PAMPs/DAMPs, such as LPS, its cystolic adaptor myeloid differentiation factor 88 (MyD88) will facilitate inducing proinflammatory cytokines, NF-κB, for instance, translocating into nuclear and transcriptionally upregulating IL-1β, IL-18, NLPR3, and sometimes GSDMD expression [38,39]. Secondly, the NOD structural domain of NLRP3 undergoes structural rearrangement through oligomerization and subsequently recruits ASC and exposes the effector domain, allowing the N-terminal structural domain of the hemoprotein to bind to the N-terminal structural domain of ASC, while the C-terminal CARD domain of the ASC protein recruits pro-caspase-1 with the same CARD domain to assemble into an inflammatory vesicle complex, and pro-caspase-1 cleaves itself to active caspase-1 [40]. The study on NLRC4 inflammasome in the fundamental biology of the inflammatory signaling complex was comprehensive with its inflammasome trigger–sensor–nucleator–adaptor–effector paradigm, and NLPC4 deficit or hyperactive was highlighted as associated with autoinflammatory diseases [24]. The formation of AIM2 inflammasome was based on the recognition of the double-strand DNA presented during cellular perturbation and pathogenic assault, leading to the secretion of IL-1bβ and IL-18 by pyroptosis [41].

## 5. Caspases Host Pyroptosis between Pathogenic Pathways and GSDMs

**Figure 1:**
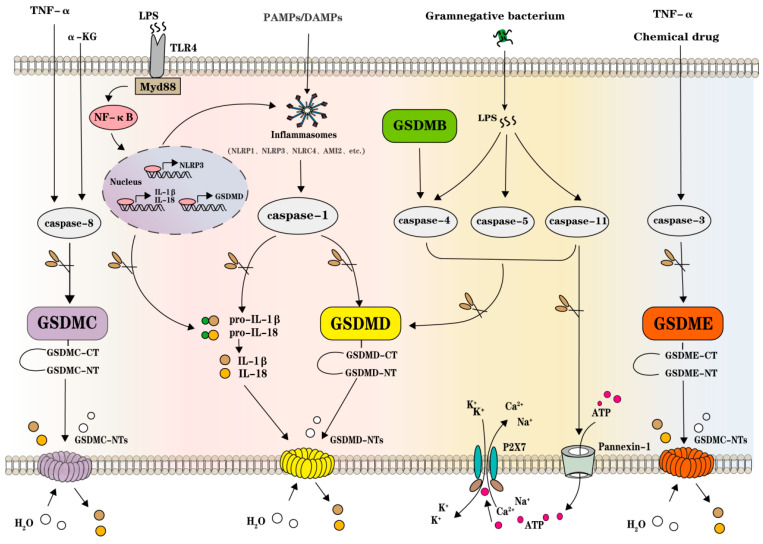
Schematic illustration of the canonical caspase-1, non-canonical caspase-4/5/11, and other caspase-3/8-mediated pyroptosis pathways. Activation of caspase-8 by TNF-α and α-KG shears GSDMC to form the pore of GSDMC-NTs, leading to the efflux of inflammatory substances IL-1β and IL-18 and the influx of H2O and the formation of cellular pyroptosis; LPS acts on TLR receptors to activate NF-κB via Myd88, NF-κB transcribes NLRP3, GSDMD, IL-1β, and IL-18, transcribed NLRP3 and GSDMD activate NLRP3 inflammatory vesicles to stimulate caspase-1 to shear GSDMD to form a pore in the cell membrane, and transcribed IL-1β and IL-18 to shear pro-IL-1β and pro-IL-18 are released outside the cell through the pore of the GSDMD-NTs, resulting in cellular pyroptosis; PAMPs/DAMPs act on inflammatory vesicles (NLRP1, NLRP3, NLRC4, AMI2, etc.) and activate caspase-1 to shear GSDMD to form GSDMD-NTs pores in the cell membrane; LPS activates caspase-4/5/11 to shear GSDMD to form a pore in the cell membrane. Caspase-11 can play a shearing role in opening the Pannexin-1 pathway, so that the intracellular ATP flows to the outside of the cell, and the effluxed ATP acts on the P2X7 receptor, which opens the P2X7 channel, resulting in the efflux of K^+^, and the inward flow of Ca^2+^Na^+^; TNF-α and Chemical drug stimulate caspase-3 to shear GSDME to form the pore of GSDMC-NTs, which allows the efflux of inflammatory substances IL-1β, IL-18 and the influx of H2O, resulting in cellular pyroptosis.

Caspase-1 mediated the canonical pyroptosis pathway, and caspase-4/5/11 was also certified in the non-canonical pyroptosis with other pathways induced by caspase-3/8. It was demonstrated in mouse macrophages that caspase-11 and caspase-1 are key targets of GSDMD [20,42]. In the canonical pyroptosis pathway, the pattern recognition receptor(PRR) is firstly stimulated by multiple PAMPs/DAMPs, and then the aggregation of inflammatory vesicles and the formation of intracellular macromolecular protein complexes are exhibited. These inflammasomes further activate pro-caspase-1 to, on the one hand, cleave inactivated pro-IL-1β and pro-IL-18 producing mature IL-1β and IL-18, and on the other hand, cut GSDMD, releasing GSDMD-NT and facilitating its oligomerization and membranal pore-forming, and the inflammatory factors of IL-1β and IL-18 are secreted extracellularly through the punched pores [43]. NLRP1, NLRP3, NLRC4, and AIM2 are all capable of assembling inflammasomes and inducing the canonical caspase-1-dependent pyroptosis pathway. LPS, as a component of the outer membrane of Gram-negative bacteria, could directly bind to non-canonical caspase-4/5/11 and lead to the cleavage of GSDMD, resulting in the generation of GSDMD-NT oligomerization and the formation of cell membrane pores causing pyroptosis [42,44]. It was worth noting that the activated caspase-11 would additionally cleave the transmembrane channel protein Pannexin-1 leaking adenosine triphosphate (ATP) outside the cell, and the released ATPs would bind to purinergic P2X7 receptors (P2X7R), causing intracellular K^+^ efflux and extracellular Na^+^ and Ca^2+^ inward flow, speeding up the cell rupture [45]. Additionally, in Yersinia pestis-infected macrophages and aged neutrophils, caspase-3/8 can also trigger pyroptosis. Active caspase-3 cleaves GSDME to produce the GSDME-NT fragments, leading to the cell swelling and rupture stressed by chemotherapy drugs or TNF-α, and α-KG- or TNF-α-induced GSDMC-mediated pyroptosis depending on the caspase-8 activation [30,46]. Studies have proven that excessive or inappropriate GSDM-mediated pyroptosis was liaised with immune defenses and multiple diseases, including tumors, metabolic disorders, aging, and other degenerative diseases, which were inherently associated with varied tissue chronic inflammatory disorders [32]. Figure 1 schematically illustrates known canonical caspase-1, non-canonical caspase-4/5/11, and other caspase-3/8-mediated pyroptosis pathways (Figure 1).

## 6. Multiple Pyroptosis Pathways Implicate DKD Pathology

**Figure 2:**
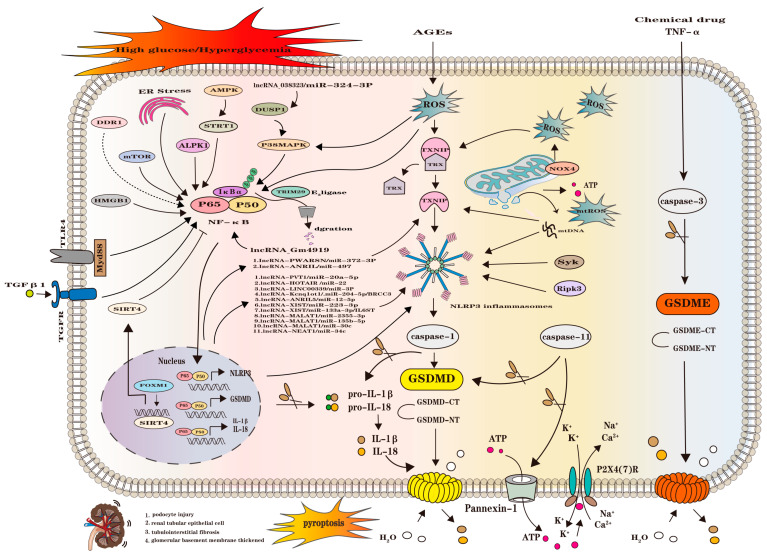
Schematic illustration of the pyroptosis pathway associated with DKD. The protein molecules HMGB1, mTOR, ALPK1, AMPK/STRT1, and DUSP1/P38MAPK can activate downstream NF-κB; lncRNA-038323/miR-324-3P; lncRNA-Gm4919 also activate downstream NF-κB; ER stress can activate NF-κB; IκBα and TRIM29 interact, and TRIM29 E3 ligase leads to IκBα duration and activation of NF-κB; TLR4 activates NF-κB through Myd88; TGFβ1 activates NF-κB through the TGFR pathway. Subsequently, activated NF-κB is translocated to the nucleus and then transcriptionally regulates NLRP3, GSDMD, IL-1β, and IL-18, which then acts on NLRP3 to activate caspase-1 to shear the GSDMD to form a pore for GSDMD-NTs, leading to the efflux of the inflammatory factors IL-1β and IL-18 and the influx of H2O and the formation of cellular pyroptosis. In addition, IL-1β and IL-18, which are transcribed by NF-κB, can directly act on pro-IL-18 and pro-IL-1β to form IL-1β and IL-18, which are released into the extracellular space. FOXM1 inhibits NF-κB activation by transcriptional activation of SIRT4, and DDR1 may activate the NF-κB/NLRP3 pathway. Nuclear transcriptional IncRNAs and miRs target TXNIP and NLRP3, the former including the Inc-PWARSN/miR-372-3P and Inc-ANRIL/miR-497 pathways; the latter encompassing lncRNA-PVT1/miR-20a-5p, lncRNA-HOTAIR/miR-22, lncRNA-LINC00339/miR-3P, lncRNA-Kcnq1ot1/.miR-204-5p/BRCC3, lncRNA-ANRIL5/miR-12-5p, lncRNA-XIST/miR-223-3p, lncRNA-XIST/miR-133a-3p/IL6ST, lncRNA-MALAT1/miR-2355-3p, lncRNA-MALAT1/miR-135b-5p, lncRNA-MALAT1/miR-30c, and lncRNA-NEAT1/miR-34c, a total of 11 pathways, and subsequent activation of caspase-1 to shear GSDMD, the pore formation, leading to cellular pyroptosis. In addition, caspase-1 shears pro-IL-1 and βpro-IL-18 to produce IL-1β and IL-18 inflammatory factors. ROS inhibits TRX/ TXNIP, causing TXNIP to stimulate NLRP3 to activate caspase-1 to shear GSDMD to form a pore for GSDMD-NTs, leading to the efflux of inflammatory factors to form cellular pyroptosis; ROS can act on p38MAPK to activate NF-κB, as well as directly activate NF-κB. NOX4 generates ROS to inhibit TRX/TXNIP; ROS can act directly on mitochondria, leading to mitochondrial damage generating mtROS and mtDNA, which act on TXNIP and NLRP3 to activate caspase-1; Syk and Ripk3 can act directly on NLRP3 to activate caspase-1 activation of caspase-1, followed by shear GSDMD involved in subsequent cellular pyroptosis. Activated caspase-11 shears GSDMD involved in the ensuing cellular focal death. In addition, caspase-11 acts on Pannexin-1 to efflux ATP, and the effluxed ATP opens the P2X4 (7) R channel to allow K^+^ efflux and Ca^2+^Na^+^ endocytosis. Chemical drugs and TNF-α activate caspase-3 to shear GSDME and form GSDME-NTs pores in the cell membrane, leading to cellular pyroptosis.

DKD is the most common microvascular lesion in diabetes with complex etiology and unclear mechanism, and the hyperglycemia induces inflammation in multiple kidney cells including podocyte and renal tubular epithelial cells, causing cell injury, tubulointerstitial fibrosis, and glomerular basement membrane thickening, which ultimately leads to renal cell injury, and the intrinsic cells of the kidney mainly include podocytes and TECs [46]. GSDMD/GSDME-mediated pyroptosis and inflammasome activation have been claimed responsible for DKD pathology, while the concrete mechanism needs to be elucidated. During acute kidney injury (AKI) and renal fibrosis induced by nondiabetic renal disease, the mechanism of pyroptosis involves classical pyroptosis in the caspase-1/GSDMD pathway as well as nonclassical pyroptosis in the caspase-4/5/11 pathway. Both signaling pathways promote the development of AKI and renal fibrosis [47]. In addition, focal death of the caspase-3/GSDME pathway can also trigger AKI by triggering focal death of renal tubular epithelial cells, leading to sub-lethal and lethal tubular injury [48,49]. In DKD, pyroptosis is closely associated with a sustained inflammatory response, and this sterile inflammation is a key feature of DKD progression and significantly influences its pathological course [50]. Figure 2 schematically shows known pyroptosis pathways involved in the physiological and pathological processes of DKD, and the following texts explain in detail (Figure 2).

## 7. TXNIP/NLRP3/Caspase-1/GSDMD Pathway

NLRP3 inflammatory vesicles are involved in the pathogenesis of DKD and promote the development of DKD under advancing hyperglycemia conditions. Lowering NLRP3 levels, demonstrated in numerous studies to mitigate renal inflammation and improve DKD, is currently critical for therapeutic intervention targeting the TXNIP/NLRP3/caspase-1/GSDMD pathway. Thioredoxin-interacting protein (TXNIP) is an upstream molecule of NLRP3 that inhibits its antioxidant activity by binding to endogenous thioredoxin (TRX). Overproduction of AGEs and mitochondrial ROS is important for activation of NLRP3 inflammatory vesicles [51]. The overproduction of ROS results in the dissociation of TXNIP from its binding protein, Trx, and subsequent TXNIP interacts with NLRP3, and this interaction leads to activation of the NLRP3 inflammasome, which in turn causes activation of caspase-1. Activated caspase-1 cleaves GSDMD, generating GSDMD-N, which forms pores in the cell membrane, thereby permitting the release of inflammatory factors such as IL-1β and IL-18, triggering pyroptosis and driving the progression of DKD [52]. In a recent study, Shahzad K. observed that specific deletion of Nlrp3 or caspase-1 in podocytes under hyperglycemic conditions could provide a protective effect against DKD by simultaneously enhancing Nlrp3 and caspase-1 function as well as weakening them in vivo, demonstrating activation of the NLRP3 inflammatory vesicle on podocyte adequacy and necessity in renal aseptic inflammation [53]. Inhibition of NLRP3 inflammatory vesicles effectively attenuates podocyte damage and reduces lipid accumulation in DK. In in vitro and in vivo experiments, treatment with knockdown of NLPR3 or MCC950 reduced the increased expression of sterol regulatory element-binding protein 1 (SREBP1) and SREBP2 as well as increased the expression of ATP-binding cassette A1 (ABCA1) in foot cells from diabetic mice and high-glucose cultures, respectively, as well as the accumulation of lipids, the activation of NF-κB p65, and the production of mitochondrial ROS [54]. In in vivo experiments, improved renal function was observed in mice by knocking out their NLRP3 gene. In addition, this knockout reduced glomerular hypertrophy and sclerosis, as well as dilatation of the thylakoid membranes and fibrosis of the interstitium. At the same time, the inflammatory response as well as the expression of transforming growth factor-β (TGF-β) and connective tissue growth factor (CTGF) were reduced in the mice. In vitro experiments led to a reduction in reactive oxygen species (ROS) production by silencing the NLRP3 gene when human kidney (HK-2) cells were subjected to a higher glycemic environment [55]. Hence, targeting NLRP3 may be a promising therapeutic approach for DKD. Overproduction of ROS was a key factor in the formation of NLRP3 inflammatory complex vesicles for inhibition of antioxidant effect by TRX-TXNIP inducing its bandage to NLRP3, which subsequently activates the NLRP3 inflammatory vesicle pathway, leading to pyroptosis [56]. The upstream of TXNIP/NLRP3 was also studied, with findings that long noncoding RNA (lncRNA) lncRNA-antisense non-coding RNA in the INK4 locus (ANRIL) directly bound microRNA (miRNA) miR-497 targeting TXNIP in high-glucose-treating HK-2 cells, and knockdown of ANRIL suppressed caspase-1 activation and pyroptosis via miR-497/TXNIP signaling [57] Another long noncoding RNA, lncRNA-PWARSN, was reported responsible for regulating TXNIP by sponging miR-372–3p-induced pyroptosis in high-glucose-treated proximal tubular epithelial cells (TECs) [58]. In high-glucose-fed Normal Rat Kidney-52E (NRK-52E) cells (a rat renal proximal TECs line), the endoplasmic reticulum stress (ERS)-related factor, inositol-requiring transmembrane kinase endoribonuclease-1α (IRE1α), unregulated miR-200a degradation and stimulated the TXNIP/NLRP3 pathway-mediated pyroptosis and renal damage [59]. Advanced glycation end products (AGEs)-induced inflammation and endothelial dysfunction could be alleviated via inhibiting ROS/NLRP3 inflammasome signaling, suggesting a therapeutic strategy in vascular complications of diabetes [60].

In a high-fat diet (HFD) and streptozotocin (STZ)-induced diabetic mouse model targeting the TXNIP/NLRP3 pathway, punicalagin, the primary polyphenol in pomegranate, protected DKD by inhibiting pyroptosis, and during this process, the nicotinamide adenine dinucleotide phosphate (NADPH) oxidase 4 (NOX4) was downregulated with mitochondrial damage restored [61]. Naringin, a naturally flavanone glycoside, ameliorated DKD by inhibiting NOX4 [62]. Tanshinone IIA (Tan IIA) is one of the main components of the root of red-rooted Salvia miltiorrhiza Bunge; it was reported recently regulating the oxidative stress and TXNIP/NIRP3 inflammasome inhibiting pyroptosis to delay the progression of DKD in db/db mice model and cultured human renal glomerular endothelial cells (HRGECs) [63]. AB-38b is a newly synthesized α,β-unsaturated carbonyl compound with biphenyl dibenzoate as precursor. An in vitro and in vivo assay showed that AB-38b possesses excellent antioxidant activity and anti-inflammatory potential, and that it was able to improve the renal function of diabetic mice, and the NLRP3 inflammasome inhibition effect of AB-38b was responsible for the renal protective effect [64]. Triptolide (TP) is a bioactive diterpene tricyclicoxide isolated from the traditional Chinese medicinal plant Treterygium wilfordii Hook F with anti-inflammatory, antioxidant, and hypoglycemic properties [65]. It improved renal function and histopathological injury of DKD mice by alleviating podocytes injury, reducing ROS through the erythroid 2-related factor 2 (Nrf2)/heme oxygenase-1 (HO-1) pathway, and weakening pyroptosis by inhibiting the NLRP3 inflammasome pathway [66]. 

## 8. NF-κB/NLRP3/Caspase-1/GSDMD Signaling Pathway

NF-κB is a transcription factor consisting of the p50 and p65 subunits and is normally bound to the IκB protein in an inhibited state. Cell signaling activates the IKK enzyme, which phosphorylates and degrades the IκB protein, releasing the NF-κB subunit. Subsequently, NF-κB enters the nucleus to activate specific genes, transcribe NLRP3, activate NLRP3, and subsequently activate caspase-1 to shear the GSDMD, which creates a pore in the cell membrane, resulting in the efflux of inflammatory factors and the formation of pyroptosis, which promotes inflammation and results in DKD [67]. Podocyte and tubular epithelial cell injuries are key factors in the pathogenesis of DKD and can be caused by the NF-κB/NLRP3 pathway. It was revealed that a subfamily of E3 ubiquitin ligases, the tripartite motif-containing 29 (TRIM29), was overexpressed in high-glucose-treated murine podocyte, and silencing TRIM29 by short hairpin RNA (shRNA) restored podocyte damage induced by pyroptosis, and mechanistic studies confirmed that TRIM29 interacts with inhibitory κB (IκBα), mediating its ubiquitination and degradation redeeming NF-κB/NLRP3 pathway activation [68]. TLR4 activation has been shown to promote inflammation, podocyte and tubular epithelial cell injury, and renal interstitial fibrosis in both in vivo and in vitro experiments under high-glucose conditions, suggesting that TLR4 is a potential therapeutic target for DKD [69]. A study using large mammal beagles as a DKD model confirmed an association between ERS and thermoprotein deposition during the pyroptosis process of DKD following the addition of 4-phenylbutyric acid (4-PBA) and BYA 11-7082 to high-glucose (HG)-treated MDCK (Martin d’Arby’s dog kidney) cells, and that ERS inhibitor 4-Phenylbutytic acid (4-PBA) and NF-κB inhibitor BYA11–7082 decreased the expression of NLRP3 and GSDMD in renal cells, suggesting that ERS alleviated the high-glucose-induced pyroptosis in Madin–Daby canine kidney (MDCK) cells through the NF-κB/NLRP3 pathway [70]. Toll-like receptor 4 (TLR4) and GSDMD were upregulated in diabetic mice and in HK-2 renal tubular epithelial cells cultured in high glucose, whereas injection of the TLR4 inhibitor TAK-242 or the NF-κB inhibitor parthenolide reduced the expression of GSDMD-NT, as well as inhibited pyroptosis and the release of IL-1β, and attenuated db/db mice’s renal tubular injury in db/db mice [71]. Synergistically, TLR4 (−/−) mice engrafted with wild-type hematopoietic cells had significantly lower serum creatinine and less tubular damage than wild-type mice reconstituted with TLR4 (−/−), suggesting that TLR4 signaling in intrinsic kidney cells played the dominant role in mediating kidney damage [72]. Under hyperglycemia conditions, the activation of the NF-κB/NLRP3 pathway by TLR4 was generally carried out by stimulating its downstream myeloid differentiation primary-response protein-88 (MyD88) in DKD. Additionally, pharmacological inhibition of MyD88 by LM8 suppressed inflammation in TECs and prevented diabetic kidney disease in experimental mice probably through the NF-κB/NLRP3 pyroptosis pathway [73].

By targeting TLR4 signaling, the high doses of 1,25(OH)_2_D_3_, 2-dodecyl-6-methoxycyclohexa-2,5-diene-1,4-dione (DMDD), berberine (BBR), Icariin (ICA), huangkui capsule (HKC), and polysaccharides from grifola frondosa (PGF) was proven to protect against tubulointerstitial fibrosis both in vitro and in vivo, and was proven to protect against tubulointerstitial fibrosis both in vitro and in vivo [74,75,76,77,78,79,80,81]. After intervention with Huangkui capsule (HKC), a modern Chinese effective medicine obtained from *Abelmoschusmanihot*, for 4 weeks, the expression levels of NLRP3, caspase-1, and IL-1β proteins were reduced in renal tissues of DKD animal models by suppressing TLR4/NF-κB signaling [82]. Artesunate (ART), an antimalarial drug possessing an anti-inflammatory effect and exhibiting a protective effect on chronic kidney diseases, was proven to ameliorate high-glucose-induced rat glomerular mesangial cell injury by suppressing the TLR4/NF-κB/NLRP3 inflammasome pathway [83]. Alpha-kinase 1 (ALPK1), a cytosolic PRR and a master regulator in inflammation, promoted tubular injury, interstitial inflammation, and renal fibrosis by inducing the IL-1β in DKD mice, and it also served as the upstream of NF-κB phosphorylation initiating the canonical caspase-1/GSDMD pyroptosis pathway [84]. Knockdown discoid domain receptor 1 (DDR1), an inflammatory regulatory protein, in high-glucose-treated HK-2 cells resulted in inhibited expression of pyroptosis-related proteins and attenuation of inflammatory factors, and the NF-κB/NLRP3 pathway was probably involved in the underlying mechanism [85]. The protective role of forkhead box M1 (FOXM1) against kidney injury by driving tubular regeneration was further mechanically explained with its transcriptionally activating sirtuin 4 (SIRT4) inhibiting the phosphorylation of NF-κB and NF-κB/NLRP3-mediated pyroptosis in podocyte [86]. Geniposide (GE) is one of the active ingredients extracted from the dried and ripe fruits of Gardenia jasminoides Ellis (Gardenia jasminoides Ellis), which has the functions of lowering blood glucose and anti-inflammation. It can inhibit the development of DKD by targeting the AMPK/SIRT1/NF-κB pathway, effectively blocking oxidative stress and the inflammatory response accompanied by thermoprotein deposition [87]. Treatment with 20(R)-ginsenoside Rg3, an active saponin isolated from ginseng (the root of Panax ginseng C. A. Meyer), in mice with DKD evaluated for 8 weeks down-regulated protein expression levels of MAPKs and NF-κB signal pathways in the kidney, improving insulin level, blood lipids, oxidative stress, and renal function [88]. By down-regulating p38MAPK and NF-κB p65 expression, the function of celastrol and hirudin in restoring renal pathological injury was experimentally confirmed combined with its effects on reducing serum creatinine and urea nitrogen as well as the excretion of urinary protein in DKD rats [89,90]. Overexpression of lncRNA NR_038323 ameliorated the interstitial fibrosis in DKD rats via miR-324–3p/dual-specificity protein phosphatase-1 (DUSP1)/p38MAPK and extracellular-regulated protein kinases (ERK) 1/2 axis, which provided new insights into the pathogenesis of DKD [91]. LncRNA-Gm4919 was found targeting NF-κB/NLRP3 inflammasome signaling and participating in the inflammation, fibrosis, and proliferation in mesangial cells under high-glucose conditions, while how Gm-4919 regulated NF-κB expression remained unknown [92]. Breviscapine (Bre), exerting a renoprotective effect, alleviated podocyte injury by decreasing α-smooth muscle actin (α-SMA) expression, increasing podocin and synaptopodin expression, and in part inhibiting NF-κB/NLRP3-mediated pyroptosis in DKD [60]. Astragaloside IV (AS-IV), an active constituent of Astragalus membranaceus, increased klotho levels in serum and renal tissues of DKD rats as well as in podocytes exposed to high glucose, reduced levels of oxidative stress and NF-κB activation, and attenuated NLRP3-mediated pyroprotein deposition in the glomeruli of DKD [93]. The plausible role of Biochanin A (BCA), an isoflavonoid, was certified in attenuating DKD by targeting the NF-κB /NLRP3 axis in both rats and NRK-52E cells [94]. Pyrroloquinoline quinone (PQQ), a naturally occurring bioactive, ameliorated renal fibrosis in DKD by inhibiting oxidative stress, phosphorylated NF-κB, the loss of mitochondrial transmembrane potential, and the NF-κB /NLRP3 pyroptosis pathway in DKD mice and high-glucose-treated HK-2 cells [95]. Swietenine (Swi), isolated from Swietenia macrophylla King, significantly improved the renal function of mice with DKD and inhibited the activation of NF-κB and the NLRP3 inflammasome and reduced inflammation by regulating the NF-κB/NLRP3/caspase-1 signaling pathway [96]. Sodium butyrate (NaB), a short-chain fatty acid, attenuates hyperglycemia-induced injury to glomerular endothelial cells (GECs) by inhibiting the typical Caspase-1/GSMD pyrolysis pathway involving the NF-κB/IκB-α signaling pathway [97]. Ginsenoside Rg1 protected podocytes from hyperlipidemia-induced damage via targeting the mammalian target of rapamycin (mTOR)/NF-κB/NLRP3 Axis by adopting L-leucine (LEU), the activator of mTOR [98]. Tanshinone IIA, which is mainly derived from Salvia miltiorrhiza Bunge (Labiatae), downregulates transforming growth factor beta 1 (TGFβ1) in addition to targeting TXNIP, thereby alleviating renal tubular epithelial cell inflammation and sepsis induced by hyperglycemia, a process in which the NF-κB pathway is involved [99]. 2,3,5,4′-tetrahydroxystilbene-2-O-β-d-glucoside (TSG), an active component extract from Polygonum multiflorum Thunb, exhibits antioxidative and anti-inflammatory effects, and ameliorated DKD in rats with the involvement of SIRT1 and TGF-β1 pathways [100]. WJ-39, an inhibitor of aldose reductase, suppressed the activation of the NF-κB pathway and the NLRP3 inflammasome to reduce the secretion of inflammatory factors in DKDA rats [101]. Liquiritigenin attenuated high-glucose-induced mesangial matrix accumulation, oxidative stress, and inflammation by suppression of the NF-κB and NLRP3 inflammasome pathways [102]. Non-pharmacologically using electro-acupuncture could suppress the high-mobility group box chromosomal protein 1 (HMGB1)/NF-κB pathway to protect DKD-induced inflammation through the suppression of NLRP3 inflammasome [103]. There were still other instances of upstream signaling of NLRP3 reported in pyroptosis of DKD and renal fibrogenesis, such as the spleen tyrosine kinase (Syk)/c-Jun N-terminal kinase (JNK)/NLRP3 pathway and receptor-interacting protein kinase-3 (RIPK3)/NLRP3 pathway [104,105]. By targeting Syk, the BAY61–3606 blocker, was proven to impede high-glucose-stimulated caspase-3-mediated HK-2 cell death [104]. 

## 9. Non-Coding RNA-RelatedNLRP3/Caspase-1/GSDMD Signaling Pathways

Depending on their length, non-coding RNAs (ncRNAs) can be classified as long non-coding RNAs (lncRNAs) and small non-coding RNAs. lncRNAs are usually larger than 200 nucleotides, whereas small ncRNAs are smaller than 200 nucleotides, and those smaller than 50 nucleotides are known as microRNAs (miRNAs). lncRNAs have a wide range of functions including cis- or trans-transcriptional regulation, nuclear structural domain organization, and protein or RNA molecule regulation, which are closely related to their subcellular location [106]. LncRNAs have a variety of functions, including cis- or trans-transcriptional regulation, organization of nuclear structural domains, and regulation of proteins or RNA molecules, which are closely related to their subcellular location. In the nucleus, lncRNAs regulate gene expression at the epigenetic and transcriptional levels, and in the cytoplasm, they influence gene expression in the post-transcriptional and translational domains [107]. Non-coding RNAs are well established as RNA transcripts do not encode proteins but regulate cell physiology and shape cellular functions, while their aberrant expressions implicate aggressive pathologies [108]. Recent studies have also revealed that lncRNAs play an important role in the regulation of cellular pyroptosis. N6-methyladenosine (m6A) served as one of the most common mRNA modifications in eukaryotes, and its methylation was disturbed by lncRNA-LINC00342 through FTO, a demethylating enzyme of m6A in DKD, in addition to LINC00667 and LNC00963 affecting FTO expression, macrophage M1-regulated DKD is mediated by m6A methylation modification by lncRNA expression [109]. LncRNA-MSC-AS1 was confirmed to promote high-glucose-mediated DKD progression in mesangial cells by regulating the miR-325/cyclin G1 (CCNG1) axis [109]. By the same token, non-coding RNA-related signaling pathways provide new ideas for DKD pathogenesis, mainly including miRNAs, lncRNAs, and circular RNAs (circRNAs); except for known non-coding RNAs functioning in biomarkers and therapeutic targets, their association with inflammasomes in pyroptosis should be reexamined [110]. In HK-2 cells, GAS5 overexpression inhibited the inflammation, oxidative stress, and pyroptosis of high-glucose-induced HK-2 cells by suppressing the expression of miR-452–5p, which provides new insights into the treatment of DKD [110]. LncRNA-NEAT 1, targeting miR-34c, regulated NLRP3 expression in DKD, providing new insights into understanding the molecular mechanism of pyroptosis in the pathogenesis of DKD [111,112]. In in vitro experiments in HK-2 cells, LncRNA-PVT1 modulated NLRP3-mediated pyroptosis by targeting miR-20a-5p in LPS-induced HK-2 cells, providing a targeting candidate for the study of DKD [113]. Knockdown of lncRNA-DLX6 antisense RNA 1 (DLX6-AS1) inhibited HK-2 cell pyroptosis by regulating the miR-223–3p/NLRP3 pathway under LPS stress, and lncRNA-XIST participated in the formation and progression of renal calculus by interacting with miR-223–3p and the NLRP3-mediated inflammatory response [114,115]. In the model of high concentration of uric acid-induced renal injury, lncRNA-HOTAIR was proven to promote endothelial cell pyroptosis by competitively binding miR-22 to regulate NLRP3 expression [116]. In HK-2 cells, Lnc-LINC00339 promoted renal tubular epithelial pyroptosis by regulating the miR-22–3p/NLRP3 axis in calcium oxalate-induced kidney stones [117]. Besides targeting TXNIP, knockdown of the lncRNA-ANRIL alleviated renal injury of uric acid nephropathy rats for the inhibited NLRP3 inflammasome activation and pyroptosis through the miR-12–5p/BRCA1-BRCA2-containing complex subunit 3 (BRCC3) axis [118]. Downregulating lncRNA-X-inactive specific transcript (XIST), targeting the miR-133a-3p/NLRP3 axis, attenuated contrast-induced nephropathy [119]. LncRNAs metastasis-associated lung adenocarcinoma transcript 1 (MALAT1) expression might enhance renal fibrosis in DKD rats and high-glucose-induced HK-2 cells via the miR-2355–3p/IL6ST axis, and further studies confirmed the MALAT1/miR-135b-5p/NLRP3 and MALAT1/miR-30c/NLPR3 signaling cascade in regulating LPS-induced inflammatory pyroptotic cell death and high-glucose-induced pyroptosis of renal tubular epithelial cells [120,121,122]. In acute kidney injury, silencing lncRNA-Kcnq1ot1 released more miR-204–5p to inhibit NLRP3 expression and its inflammasome activation, while modulating the Kcnq1ot1/miR-486a-3p/NLRP3 and Kcnq1ot1/miR-506–3p/NLRP3 regulatory axis facilitated podocyte-targeted treatment for renal inflammatory diseases [123,124]. Another miRNA targeting the 3′untranslated region of NLRP3 mRNA, miR-10a/b, was proven to function as a negative regulator of the NLRP3 inflammasome, inhibiting assembly of the NLRP3 inflammasome and decreasing caspase-1-dependent release of pro-inflammatory cytokines [125]. CircRNA, circ_0004951, was significantly unregulated in DKD patients and high-glucose-treated HK2 cells, and knockdown of circ_0004951 suppressed pyroptosis by inhibiting miR-93–5p, which was responsible for NLPR3 expression [126]. Pyroptosis in macrophages interacted with TECs regulating the progression of renal fibrosis, and circACTR2 regulated macrophage inflammation, epithelial–mesenchymal transition, and the development of renal fibrosis by activating NLRP3 inflammasome via sponging miR-561 [127]. By using circRNA microarray analysis, a series of dysregulated circRNAs were profiled in glucose-stressed HK-2 cells, and the upregulated circACTR2 was proven to be involved in inflammation and pyroptosis [128]. Further studies claimed that the circACTR2 functioned in M2 macrophages and repressed miR-200c expression, and knockdown circACTR2 boosted miR-200c expression, reduced Yes Associated Protein (YAP) level, and lowered M2 macrophages in the obstructed kidney [129]. Nevertheless, in human renal mesangial cells, circACTR2 aggravated under high-glucose conditions and activated the miR-205–5p/high-mobility group AT-hook 2 (HMGA2) axis, providing a novel target for the diagnostic and therapeutic potential of DKD treatment [129].

## 10. ATP/P2X4(7)/NLRP3/Caspase-1/GSDMD Signaling Pathway

P2X7 receptors are expressed in many types of cells including stem, blood, glial, neural, ocular, bone, dental, exocrine, endothelial, muscle, renal, and skin cells, and the P2X7/NLRP3 pathway plays an essential role in amplifying inflammation via an ATP feedback loop, during which the ATP releases from dying cell, acting as a “danger signal” to further amplify the inflammatory signal by the hyperglycemia insult [130,131]. ATP is released via ATP-permeable channels, together with other metabolites, from injured TECs, and P2X4- or P2X7-positive macrophages underwent pyroptosis after unilateral ureteral obstruction with ATP, directly inducing pyroptosis by macrophages [132]. In nephrocalcinosis, crystal-induced extracellular ATP upregulation via the membrane P2X7R promotes ROS generation, thereby activating NLRP3 inflammasome-mediated IL-1β/18 maturation and GSDMD cleavage [133]. P2X4 confirmed increased expression in renal TECs in patients with ND, and ATP/P2X4 signaling was activated in renal interstitial inflammation and correlated to the NLRP3-mediated pyroptosis [134,135]. In podocytes of glomeruli, DKD rats were shown with a dramatic increase in ATP-mediated intracellular calcium signaling, and the pharmacological use of the inhibitors of renal P2X4 and P2X7 facilitated the transition from metabotropic to ionotropic composition, alleviating intracellular calcium homeostasis [136]. In diabetic patients, P2X7R expression was associated with severe mesangial expansion, impaired glomerular filtration, and increased interstitial fibrosis, and P2X7R deficiency, or using a P2X7R inhibitor (AZ11657312) could reduce the renal macrophage accumulation [137]. The application of the nonselective P2X4 and P2X7 antagonist, suramin, prevented the development of DKD by inhibiting NLRP3 inflammasome activation in mice models [138]. Similarly, esculin reduced P2X7 levels in DKD rats and restored mitochondrial function via glycolysis substrates and β-oxidation, which might associate its molecular interact with NLRP3 inflammatory pyroptosis [139]. P2X7R and NLRP3 inflammasome were involved in the pathogenesis of DKD, and *Ophiocordyceps sinensis* (ACOS) could effectively inhibit the high expression of P2X7R and the activation of NLRP3 inflammasome, which might contribute to the therapeutic effects of *Ophiocordyceps sinensis* in DKD [140]. Except for caspase-1, caspase -11, or caspase-4 (the homolog of caspase-11 in humans), knockout in diabetic mice and cultured human/mouse podocyte might blunt the reduced expression of podocyte markers nephrin and podocin, loss and fusion in the podocyte foot process, increased inflammatory cytokines NF-κB, IL-1β, and IL-18, macrophage infiltration, and glomerular matrix expansion due to the inhibition of GSDMD-mediated pyroptosis [141]. As previously mentioned, pannexin-1 and P2X7 also served as the downstream of caspase-11, mediating the non-canonical inflammasome and pyroptosis [45].

## 11. Caspase-3/GSDME Signaling Pathway

The newly identified caspase-3/GSDME-dependent pyroptosis signaling pathway and its physiological and pathological role in DKD have been focused on and studied, and more direct research efforts should be conducted in this area. Chemical drugs and TNF-a activate caspase-3. Subsequently, activated caspase-3 cleaves GSDME to generate GSDME-N and GSDME-C fragments, and GSDME-N perforates the plasma membrane to form permeable pores. In addition, pro-caspase-1 leads to cleavage of pro-IL-1β, resulting in the production of mature biologically active mIL-1β. These mature IL-1β molecules are released through the pore, causing inflammatory damage in DKD [16,142]. In the kidney of structural and functional disorders, the expression of caspase-3 and GSDME protein in the renal cortex were significantly upregulated, and knockdown GSDME by injection with adeno-associated virus (AAV)-shGSDME reduced kidney damage and renal cell pyroptosis, providing an important basis for clinical therapeutic studies [16]. In the renal tubular cell, the cell fate was determined by GSDME expression and GSDME-NT generation response by TNFα under the condition of oxygen–glucose–serum deprivation, and deletion of caspase-3 or GSDME alleviated renal tubule damage and inflammation, preventing hydronephrosis and kidney fibrosis [143]. It was demonstrated that the chemotherapeutic drug cisplatin or doxorubicin mediated the cleavage of GSDME in cultured human renal TECs in a time- and concentration-dependent manner; during this process the caspase-3 activation was indispensable [144]. A class of GSDME-derived inhibitors containing the core structure of DMPD or DMLD was developed, and Ac-DMPD-CMK and Ac-DMLD-CMK could directly bind to and inhibit the catalytic domains of caspase-3, blocking the cleavage function on GSDME and preventing pyroptosis in the liver, implying a therapeutic role in DKD Treatment with Ac-DMLD-CMK, a peptide targeting caspase-3/GSDM [145]. E signaling in mice inhibited caspase-3 and GSDME activation, alleviated the deterioration of renal function, attenuated TECs injury, and reduced inflammatory cytokine secretion in vivo [144]. The GSDME-deficient mice and human TECs were employed to prove that caspase-3/GSDME-triggered pyroptosis and inflammation contribute to acute kidney injury, and treatments targeting GSDME could be a new insight into DKD [146]. 

## 12. Targeting Pyroptosis on DKD Treatment

Pyroptosis is involved in DKD guiding new directions and therapeutic implications, and by targeting pyroptosis, investigational therapeutic compounds have been reported and tested in animal or cell models to repress renal cell death. By targeting the NLRP3 inflammasome or other signals, several particles were proven effective in DKD treatment with unrevealed mechanisms, and they were systematically reviewed in 2021 [6]. In the kidney of db/db mice, the NLRP3 inflammasome activity was repressed by curcumin [147]. Dihydroquercetin (DHQ) possessed kidney protection effects including attenuating urine microalbumin excretion, hyperglycemia, and lipid metabolism disorders and mitigating renal histopathological lesions on dn by suppressing ROS and NLRP3 inflammasome [148]. Treatment with MCC950, an NLRP3 inflammasome-specific inhibitor, improved renal function, attenuated albuminuria, mesangial expansion, podocyte loss, as well as glomerular lipid accumulation in db/db mice [57]. Ginsenoside Rg5 (Rg5) attenuated renal injury in diabetic mice by inhibiting oxidative stress and NLRP3 inflammasome activation to reduce inflammatory responses [149]. Sarsasapogenin (SAR), a steroidal sapogenin, could markedly ameliorate diabetic kidney disease in rats via inhibition of NLRP3 inflammasome activation and AGEs–RAGE interaction [150], after supplementation with polysaccharides from Armillariella tabescens mycelia (AT), modulating the intestinal microbiota and inflammatory reaction in DKD mice [151]. Clinically using a low dose of pioglitazone (PIO) was an effective, safe, and inexpensive method of reducing proteinuria in type 2 diabetic patients with nephropathy [152]. Maresin 1 (MAR 1), one of the potent anti-inflammatory lipid facilitators achieved from n-3 poly-unsaturated fatty acids, could inhibit NLRP3 inflammasome, TGF-β1 in GMCs showing protective effects on DKD by mitigating the inflammation and early fibrosis [153]. FL-926–16, a carnosinase-resistant and bioavailable carnosine derivative, was effective at preventing the onset of DKD and halting its progression in db/db mice by quenching the AGE precursors reactive carbonyl species (RCS), thereby reducing the accumulation of their protein adducts and the consequent inflammatory response [154]. As mentioned earlier in this article, we summarized the known DKD therapeutic particles and arranged them by targeting pathways. Table 1 shows the mentioned particles interacting with molecules therapeutically regulating or functioning in GSDMD- or GSDME-induced pyroptosis pathways in DKD treatment (Table 1). Notably, all these targets are tested in the DKD animal model or high-glucose-induced cell model, and the clinical translation needs to be further studied. Meanwhile, the protective effects of the drug alone and the combination may be different, and the optimal dosage and composition of the mixture need to be further developed (Table 2). Additionally, the potential clinical application of these drugs must consider not only their efficacy in reducing pyroptosis but also their safety profiles and possible side effects in humans. Rigorous clinical trials are essential to ascertain their therapeutic potential and to determine the most effective and safe therapeutic strategies for treating diabetic nephropathy through the modulation of pyroptosis pathways.

Undoubtedly, side effects of the medicine are unavoidable, inducing hypoglycemia, hypokalemia, acute pancreatitis, etc., while non-pharmacological means of treatment are recommended, especially in the early stages of DKD, mainly focused on exercise, electrostimulation, and dietary therapeutic strategies. Here, the research progress of non-pharmacological treatments affecting pyroptosis further improving DKD was elucidated to provide basic mechanism theoretical fundaments and applicable data support. Studies have investigated the effects of different exercise parameters such as aerobic/endurance and resistance exercises on DKD to provide effective training guidelines for improving diabetic DKD. A systematic review revealed that aerobic exercise (AE) improved oxidative indicators of MDA and SOD in DKD patients [155]. In db/db mice with DKD, seven consecutive weeks of one-hour-a-day moderate-intensity training reduced renal function, morphology, and caspase-3 and caspase-8 activity, while improving SOD expression and reducing oxidative damage, implying that pyroptosis may be attenuated [156]. Aerobic exercise (AE) is an effective tool for the prevention and treatment of DKD, and its intervention mechanism is not only through the inhibition of ROS but also based on the NLRP3 inflammatory vesicles. Eight-week-AE training alleviated renal injury in db/db mice by inhibiting NOX4-mediated NLRP3 inflammasome activation by the NOX4/ROS/NF-κB signaling pathway [157]. Similarly, 8-week AE of treadmill attenuated excessive apoptosis, restored autophagy in the renal cortex, and inhibited the development of mitochondrial morphological abnormalities in proximal tubular cells, which was accompanied by restoration of AMPK activity and inhibition of the mTOR pathway [158]. Resistance exercise (RE) also has irreplaceable advantages in improving glucose and lipid metabolism in muscle tissue due to its unique exercise characteristics. Moreover, appropriate RE can improve muscle strength, blood glucose control, insulin sensitivity, and vascular endothelial function in DKD mice. It was proven that RT could regulate renal renin–angiotensin system (RAS) components and inflammatory mediators in diabetic rats by shifting the balance of renal RAS toward angiotensin-converting enzyme (ACE2)/angiotensin (Ang) 1–7 axis and mitigating the high levels of IL-10 and IL-1β [159]. Different forms of exercise, such as AE and RE, can effectively improve DKD, providing rehabilitation methods beyond drug therapy for DKD patients. However, the detailed molecular mechanisms, especially based on pyroptosis, by which exercise exerts beneficial effects in suppressing DKD have not yet been fully elucidated. Last but not least, electroacupuncture and diet therapy also plays important roles in the treatment and prevention of DKD. Electroacupuncture (EA) can protect DKD-induced inflammation by inhibiting NLRP3 inflammasome. In the DKD mouse model, EA inhibited HMGB1 targeting the NLRP3/NF-κB pathway to increase the anti-inflammatory effect [103]. Diet therapy is also an effective way to improve DKD. A high-carbohydrate, high-fiber, and low-fat diet can reduce the weight of diabetic patients and prevent diabetes in high-risk groups [160]. Subsequently, Li Y.J. found that dietary fiber can prevent diabetic kidney disease by regulating intestinal microflora and producing short-chain fat bacteria, and the activation of G protein-coupled receptors GPR43 and GPR109A claimed responsibility for the effects [161]. In addition, probiotics can improve blood glucose control in patients with diabetic kidney disease, and vitamin D treatment is beneficial to prevent and improve some diabetic complications. Vitamin D supplementation can reduce the incidence of T2DM and improve blood glucose control by increasing insulin secretion, reducing insulin resistance, and reducing inflammation [162]. So far, it is regrettable that there is less evidence on diet affecting the pyroptosis pathway ameliorating DKD, and further related studies need to be conducted urgently.

## 13. Conclusions

This paper has reviewed the mechanisms of pyroptosis and its canonical, non-canonical, and caspase-3/8-mediated inflammatory pyroptosis pathways to the pyroptotic pathogenesis in DKD.The pathogenesis of DKD-related pyroptosis was proven associated with known signaling pathways activated by ROS, TLR4, lncRNAs, and P2X4/7 channels, and mediated by caspase-1, NLRP3, GSDMD, GSDME, caspase-4/5/11, caspase-3/8, and more exact mechanisms and upstream targets need to be well elucidated. (1) Exploration of pyroptosis-associated inflammatory vesicles. Investigating additional pathways and discovering new molecules or drugs that modulate these vesicles represents the most promising direction. This research could lead to novel therapeutic targets and strategies for DKD treatment. (2) Investigation of exercise effects on DKD. While exercise shows potential in preventing and treating DKD, understanding its molecular interactions with pyroptosis pathways is crucial. Current research predominantly focuses on renal pathology, necessitating deeper investigations into these interactions to optimize exercise-based interventions. (3) Inhibition of GSDME-induced pyroptosis. Addressing this gap in research, particularly through combined non-pharmacological exercise treatments, offers significant promise for alleviating DKD progression. Exploring dose–response relationships of pharmacological and non-pharmacological strategies targeting pyroptosis is essential for refining treatment approaches. These priorities are outlined to enhance our knowledge and therapeutic strategies for DKD, addressing critical gaps and paving the way for innovative treatments.

## Figures and Tables

**Table 1:** Non-coding RNA-related pathways.

Upstream	Non-Coding RNA	Downstream
	lncRNA-038323/miR-324-3p	DUSP1/p38MAPK/ERK1/2
	lncRNA-Gm4919	NF-κB
**Nucleus**	IncRNA-PWARSN/miR-372-3P IncRNA-ANRIL/miR-497	TXNIP
**Nucleus**	lncRNA-PVT1/miR-20a-5p lncRNA-HOTAIR /miR-22 lncRNA-LINC00339/miR-3P lncRNA-Kcnq1ot1/miR-204-5p/BRCC3 lncRNA-ANRIL5/miR-12-5p lncRNA-XIST/miR-223-3p lncRNA-XIST/miR-133a-3p/IL6ST lncRNA-MALAT1/miR-2355-3p lncRNA-MALAT1/miR-135b-5p lncRNA-MALAT1/miR-30c lncRNA-NEAT1/miR-34c	NLRP3 inflammasomes

DUSP1, dual-specificity phosphatase 1; ERK1/2, extracellular-regulated protein kinases; NF-κB, nuclear factor-κB; TXNIP, thioredoxin-interacting protein.

**Table 2:** Particles therapeutically regulating pyroptosis in DKD.

Pyroptosis Pathway or Component	Treatment Agents	Results (Compared with Controls)	Potential Clinical Translation and Efficacy	Reference
TXNIP/NLRP3/ caspase-1/GSDMD pathway	Punicalagin	↓BUN, ↓CREA, and ↓UACR	alleviating DKD by reducing inflammation and pyroptosis	[61]
	Naringin	↓Scr, and ↓BUN	improving renal function in DKD by reducing oxidative stress and inflammation	[62]
	Tan IIA	↓Scr, ↓BUN, and ↓albuminuria	reducing renal tubular epithelial cell inflammation and pyroptosis in DKD	[63]
	AB-38b	↓Scr, and ↓BUN	improving renal function and reducing fibrosis in DKD by targeting oxidative stress and NLRP3 inflammasome	[64]
	TP	↓Scr, and ↓BUN	alleviating podocyte injury and improving renal function in DKD by reducing oxidative stress and pyroptosis	[65]
NF-κB/NLRP3/ caspase-1/GSDMD pathway	4-PBA	↓ER stress, and ↓pyroptosis	alleviating ER stress-induced pyroptosis in diabetic nephropathy	[70]
	BYA11–7082	↓tubular injury score	reducing NLRP3-mediated pyroptosis in DKD	
	TAK-242	↓caspase-1, ↓GSDMD-NT, ↓IL-18, and ↓IL-1β	alleviating TLR4-mediated GSDMD-induced pyroptosis in DKD	[71]
	LM8	↓Scr, ↓BUN, and ↓ACR	therapeutic inhibition of MyD88-mediated renal inflammation in diabetes	[77]
	HKC	↓Scr, ↓BUN, ↓UAlb, and ↓Alb	therapeutic intervention in renal tubular and inflammation in DKD	[82]
	PGF	↓Scr, and ↓BUN	can be developed as a complementary treatment for diabetic nephropathy, potentially improving glucose control and renal protection alongside existing therapies	[79]
	1,25(OH)2D3	↓Scr, and ↓BUN	consideration for anti-inflammatory and antifibrotic therapies in renal diseases	[76]
	DMDD	↓Scr, and ↓BUN	treating diabetic nephropathy by targeting inflammation and improving kidney function and metabolic parameters	[75]
	ICA	↓Scr, ↓BUN, and ↓UAlb	mitigating renal inflammation and oxidative stress	[68]
	BBR	↓24h-urinaryproteinlevel, ↓Scr, and ↓BUN	reducing renal injury and inflammation and benefitting podocyte survival in DKD	[74]
	ART	↓Scr, ↓BUN, and ↓UAlb	reducing inflammation, oxidative stress, and extracellular matrix accumulation in DKD	[83]
	GE	↓Scr and, ↓BUN	reducing kidney inflammation and dysfunction in DKD	[87]
	Cat	↓Scr and, ↓BUN	reducing inflammation, oxidative stress, and preventing kidney damage in DKD	[87]
	20(R)-Rg3	↓Scr and, ↓BUN	alleviating symptoms of diabetic nephropathy by improving renal function and metabolic profiles.	[88]
	celastrol	↓Scr, ↓BUN, and ↓urinary protein	protecting kidneys in diabetic nephropathy by reducing inflammation and delaying renal damage	[89]
	hirudin	↓Scr, and ↓BUN	protecting against kidney damage in diabetic nephropathy by inhibiting inflammation and preventing podocyte apoptosis	[90]
	AS-IV	↓Scr, ↓BUN, and ↓UACR	improving renal function and reducing podocyte damage in DKD models	[93]
	BCA	↓Scr, and ↓BUN	protecting against renal injury and fibrosis	[94]
	PQQ	↓Scr, and ↓BUN	reducing inflammatory cytokine levels associated with DKD and protecting against oxidative stress-induced renal damage	[95]
	SWI	↓Scr, ↓BUN, and ↓UACR	reducing inflammatory markers associated with DKD progression and protecting renal function	[96]
	Ginsenoside Rg1	↓Scr, ↓BUN, and ↓UACR	alleviating renal damage induced by hyperlipidemia in DKD	[98]
	TSG	↓Scr, and ↓BUN	improving renal function, reducing proteinuria in diabetic patients, and enhancing antioxidant defenses in diabetic conditions	[100]
	WJ-39	↓ACR, and ↓Scr	improving renal function and reducing fibrosis in DKD patients	[101]
ATP/P2X4 (7)/ NLRP3/caspase-1/ GSDMD pathway	AZ11657312	↓Scr, and ↓AER	reducing renal macrophage accumulation and fibrosis and mitigating glomerular mesangial expansion and preserving renal function	[137]
	Suramin	↓UACR, and ↓AER	preventing or slowing the progression of early-stage DKD	[138]
	ACOS	↓Scr	preventing or attenuating kidney damage in diabetes through anti-inflammatory and podocyte-protective effects	[140]
Caspase-3/GSDME pathway	Ac-DMPD-CMK	↓ALT/AST, and ↓LDH	advancement as specific caspase-3 inhibitors for clinical use and alleviating liver injury	[145]
	Ac-DMLD-CMK			
NLRP3	Curcumin	↓IL-1β, ↓caspase-1, and ↓NLRP3 level	a potent antifibrotic agent that inhibits NLRP3 activity to protect kidney	[147]
	DHQ	↓ECM, and ↓mesangial matrix expansion	reducing urine microalbumin excretion, hyperglycemia, and lipid metabolism disorders in DKD	[148]
	Rg5	↓insulin levels, ↓serum creatinine, ↓serum urea, ↓and serum UA	improving renal function and histopathology in diabetic mice and reducing kidney inflammation	[149]
	SAR	↓albuminuria, ↓kidney weight index, ↓serum uric acid, and ↓ECM	improving renal function and morphology in diabetic rats, and reducing renal inflammation and fibrosis	[150]
	AT	↓renal function-related indices, ↓LPS, ↓IL-1β, and ↓IL-18	improving intestinal health, which could translate to broader metabolic benefits in diabetic patients	[151]
	MAR 1	↓ROS, ↓NLRP3, ↓caspase-1, ↓IL-1β, and ↓TGF-β1 level	mitigating inflammation and fibrosis in DKD, potentially preventing disease progression	[153]
	FL-926–16	↓creatinine; ↓albuminuria, and ↓proteinuria	preventing onset and halting progression of DKD	[154]

↓: downregulate; DKD: diabetic kidney disease; BUN: blood urea nitrogen; CREA: serum creatinine; UACR: urine albumin-to-creatinine ratio; Scr: screatinine; Alb: albumin; Tan IIA: tanshinone IIA; TP: triptolide; 4-PBA: 4-phenylbutytic acid; HKC: huangkui capsule: UAlb: urinary albumin; GE: geniposide; Cat: catalpol; PQQ: pyrroloquinoline quinone; SWI: swietenine; TSG: tetrahydroxy stilbene glucoside; ACOS: ophiocordyceps sinensis; LDH: lactate dehydrogenase; ALT: alanine aminotransferase; AST: aspartate aminotransferase; ICA: icariin; DKD: diabetic kidney disease; PGF: polysaccharides from grifola frondose; DMDD: 2-dodecyl-6-methoxycyclohexa-2,5-diene-1,4-dione; BBR: berberine; ART: artesunate; GE: geniposide; AS-IV: astragaloside IV; BCA: biochanin A; DHQ: dihydroquercetin; ECM: extracellular matrix; PIO: pioglitazone; Rg5: ginsenoside Rg5; SAR: sarsasapogenin; AT: armillariella tabescens mycelia; MAR 1: maresin 1.

## Abbreviations

Caspases, cysteine-dependent aspartate-specific proteases; GSDMs, gasdermins; IL-1β, interleukin-1β; IL-18, interleukin-18; DKD, diabetic kidney disease; ESKD, end-stage renal disease; NLRP3, NOD-like receptor thermal protein domain-associated protein 3; ROS, reactive oxygen species; LPS, lipopolysaccharide; GSDME-N, gasdermine-N-terminal suppression domains; CARD, caspase recruitment domain; TNF-α, tumor necrosis factor-alpha; Pro-caspase-1, protein pro-cysteinyl aspartate-specific proteinase-1; PRR, pattern recognition receptor; DAMPs, damage-associated molecular patterns; PAMPs, pathogen-associated molecular patterns; ASC, apoptosis-associated spot-like protein; PYD, pyrin domain; NOD, nucleotide-binding and oligomerization domain; LRRs, leucine-rich repeats; TLRs, Toll-like receptors; LPS, lipopolysaccharide; P2X7, purinergic receptorsp2x7; α-KG, α-ketoglutarate; α-SMA, α-smooth muscle actin; SIRT1, silent mating-type information regulation 1; TXNIP, thioredoxin-interacting protein; Trx, thioredoxin; HG, high glucose; HK-2, human renal tubular epithelial cells; NF-κB, nuclear factor-κB; STZ, streptozotocin; MyD88, myeloid differentiation factor 88; PGF, polysaccharides from grifola frondose; MAPK, mitogen-activated protein kinases; AGEs, advanced glycosylation end products; INS, insulin; DUSP1, dual-specificity phosphatase 1; ERK1/2, extracellular-regulated protein kinases; RTC, renal tubular cell; TEC, tubular epithelial cells; DHQ, dihydroquercetin; AE, aerobic exercise; AMPK, adenosine 5‘-monophosphate (AMP)-activated protein kinase; TET, treadmill exercise; T2DM, type 2 diabetes mellitus; RT, resistance exercise; ET, endurance exercise; SDT, spontaneously diabetic tori; EA, electroacupuncture; HMGB1, high-mobility group box chromosomal protein 1.
